# HDL-Cholesterol and Triglycerides Dynamics: Essential Players in Metabolic Syndrome

**DOI:** 10.3390/antiox14040434

**Published:** 2025-04-03

**Authors:** Sebastià Alcover, Lisaidy Ramos-Regalado, Gabriela Girón, Natàlia Muñoz-García, Gemma Vilahur

**Affiliations:** 1 Research Institute Sant Pau (IR SANT PAU), 08041 Barcelona, Spain; salcover@santpau.cat (S.A.); lramosr@santpau.cat (L.R.-R.); ggiron@santpau.cat (G.G.); nmunoz@santpau.cat (N.M.-G.); 2 Facultat de Biologia, Universitat de Barcelona, 08028 Barcelona, Spain; 3 Facultat de Biociències, Universitat Autònoma de Barcelona, 08193 Barcelona, Spain; 4 Centro de Investigación Biomédica en Red Enfermedades Cardiovasculares, Instituto de Salud Carlos III, 28029 Madrid, Spain

**Keywords:** triglycerides, HDL, metabolic syndrome, lipid metabolism, reverse cholesterol transport, antioxidant activity, insulin resistance, atherosclerosis, cardiovascular disease

## Abstract

Metabolic syndrome (MetS) is a complex cluster of interrelated metabolic disorders that significantly elevate the risk of cardiovascular disease, making it a pressing public health concern worldwide. Among the key features of MetS, dyslipidemia—characterized by altered levels of high-density lipoprotein cholesterol (HDL-C) and triglycerides (TG)—plays a crucial role in the disorder’s progression. This review aims to elucidate the intricate interplay between HDL-C and TG within the context of lipid metabolism and cardiovascular health, while also addressing the detrimental impact of various cardiovascular risk factors and associated comorbidities. The dynamics of HDL-C and TG are explored, highlighting their reciprocal relationship and respective contributions to the pathophysiology of MetS. Elevated levels of TGs are consistently associated with reduced concentrations of HDL-C, resulting in a lipid profile that promotes the development of vascular disease. Specifically, as TG levels rise, the protective cardiovascular effects of HDL-C are diminished, leading to the increased accumulation of pro-atherogenic TG-rich lipoproteins and low-density lipoprotein particles within the vascular wall, contributing to the progression of atheromas, which can ultimately result in significant ischemic cardiovascular events. Ultimately, this paper underscores the significance of HDL and TG as essential targets for therapeutic intervention, emphasizing their potential in effectively managing MetS and reducing cardiovascular risk.

## 1. The Metabolic Syndrome: Epidemiology and Introduction

The earliest associations between comorbidities, including visceral obesity, hypertension, and atherosclerosis, among others, date back to the eighteenth century [1]. However, it was not until the twentieth century that the term metabolic syndrome (MetS) was coined [2] and was defined as the cluster of at least three out of the following five criteria: a high level of abdominal obesity along with visceral fat accumulation (measured by waist circumference), elevated triglyceride (TG) levels, low high-density lipoprotein cholesterol (HDL-C) levels, elevated blood pressure, and increased hyperglycemia and/or hyperinsulinemia [2]. MetS is primarily driven by increased consumption of high-calorie/low-fiber food and a decline in physical activity caused by mechanized transportation and sedentary lifestyles. Monitoring the prevalence of MetS across diverse populations is crucial due to its strong association with an increased risk of developing type 2 diabetes mellitus [2], cardiovascular disease (CVD) [3], and chronic kidney disease [4]. It is estimated that approximately 1.5 billion people (20% of the global population) have MetS [5], in line with the observations of a recent meta-analysis (ranging between 12.5 and 31.4%) [6]. From a financial perspective, the economic burden of MetS, encompassing healthcare expenses and the loss of potential economic productivity, amounts to trillions of dollars [7]. Interestingly, contrary to expectations, there is no significant association between the increase in MetS prevalence and a country’s economic status. This trend is observable in African nations where rapid urbanization and the high cost of healthy foods promote unhealthy lifestyle choices, and the prevalence of MetS is increasing [5].

In the following review, we comprehensively examine the role of two key components of MetS, HDL and TG. We first overview the biogenesis of HDL and TG, discuss their role in the cardiovascular system, and explore the detrimental impact of cardiovascular risk factors. We also examine the dynamic connection between HDL and TG particles and their contribution to MetS progression to finally examine potential therapeutic strategies.

## 2. TG and HDL Biogenesis

Lipid metabolism is a complex and intricately regulated process that encompasses the synthesis, breakdown, and transport of lipids, which are essential for energy homeostasis, cellular signaling, and maintenance of membrane structure. These lipids can originate from dietary sources, referred to as the exogenous pathway, or can be synthesized by cells via the endogenous pathway [8].

**Figure 1:**
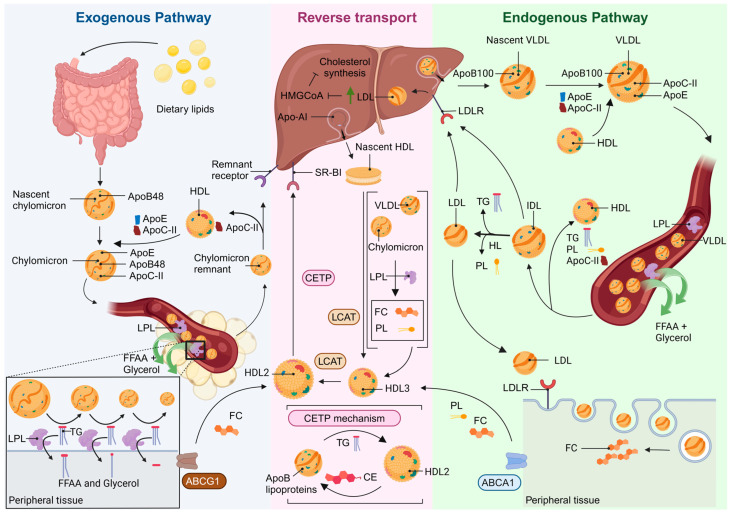
Overview of lipid metabolism pathways. The exogenous pathway starts in the small intestine, where dietary lipids are hydrolyzed, absorbed, and reassembled into nascent chylomicrons. These lipoproteins interact with HDL and acquire apoE and apoC-II. Mature chylomicrons transport TG and cholesterol to peripheral tissues, where LPL hydrolyses TG, releasing FFAA for energy or storage. The chylomicron remnants can interact with HDL and are taken up by the liver. The endogenous pathway is triggered during fasting, with the liver synthesizing VLDL to distribute TG and cholesterol. Nascent VLDL particles receive apoE and apoC-II from HDL’s interactions. Mature VLDL is progressively metabolized into IDL and LDL, which return directly to the liver for degradation or deliver cholesterol to tissues by LDLR binding and endocytosis. HDL particles mediate reverse cholesterol transport, shuttling cholesterol from peripheral tissues to the liver for its excretion. ApoA-I is synthesized in the liver and acquires free cholesterol and PL as a result of LPL activity or via ABCA1 transporter from peripheral cells. LCAT esterifies free cholesterol on the HDL surface. As HDL matures, it engages with ABCG1 transporters from peripheral cells to acquire free cholesterol and interacts with CETP to transfer TG in exchange for CE with apoB-containing lipoproteins. Then, HDL can either deliver cholesterol directly to the liver via the SR-BI receptor or indirectly by via CETP apoB-containing lipoproteins, which are then cleared by the liver. Apo: apolipoprotein; TG: triglycerides; LPL: lipoprotein lipase; FFAA: free fatty acid; VLDL: very-low-density lipoproteins; IDL: intermediate-density lipoproteins; LDL: low-density lipoproteins; HDL: high-density lipoproteins; LDLR: LDL receptor; PL: phospholipids; LPL: lipoprotein lipase; ABCA1: ATP-binding cassette protein A1; LCAT: lecithin–cholesterol acyltransferase; ABCG1: ATP-binding cassette protein G1; CETP: cholesteryl ester transfer protein; CE: cholesterol esters; SR-BI: scavenger receptor class B type I; Green arrow: increase. Created in BioRender.com.

The exogenous pathway begins in the small intestine where dietary lipids are absorbed and metabolized (Figure 1). During digestion, lipases hydrolyze the ester bonds in TG, leading to the production of free fatty acids (FFAA) and 2-monoacylglycerol. These digestive products are then emulsified by bile acids and phospholipids (PL) in the intestinal lumen, to form micelles [9]. Upon absorption into the enterocyte, FFAA and 2-monoacylglycerol are used by intestinal cells to synthesize TG, while cholesterol is converted into cholesterol esters (CE). These molecules, along with PLs and apolipoprotein (apo) B48, are subsequently assembled into nascent chylomicrons [10,11]. Nascent chylomicrons circulate through the bloodstream, acquiring apoC-II and apoE from HDL. As they travel to various tissues, they pass through capillaries, where apoC-II activates lipoprotein lipase (LPL), an enzyme also stimulated by insulin. LPL hydrolyzes the TG content of chylomicrons, producing glycerol and FFAA. The FFAA are then taken up by cells for either oxidation or re-esterification into TGs [12,13]. As TGs are depleted from chylomicrons, smaller chylomicron remnant particles are formed. ApoC-II is transferred back to HDL, while apoE is recognized by remnant receptors on hepatocytes. This interaction facilitates the uptake of chylomicron remnants by the liver, where they are further degraded [14].

The endogenous lipid transport pathway is activated during fasting, triggering de novo cholesterol and TG synthesis in the liver [15]. As such, lipids are packaged and secreted as nascent very low-density lipoproteins (VLDL) characterized by the presence of surface apoB-100. Like chylomicrons, VLDL particles acquire apoC-II and apoE from HDL. Upon reaching the vasculature, LPL activation in capillary beds leads to the hydrolysis of TG from VLDL and the subsequent release of FFAA and glycerol [16]. A portion of the TG, PLs, and apoC-II is transferred to HDL, which converts VLDL into denser intermediate-density lipoproteins (IDL). Low-density lipoproteins (LDL), chylomicron remnants, and some IDL can be taken up by the liver via LDL receptors (LDLR) that recognize apoB100 and apoE on the lipoprotein surface. Moreover, TGs contained in the remaining IDL fraction are hydrolyzed by hepatic lipase, generating LDLs, which are predominantly composed of CE. LDL binds to LDLRs, which are present in the cell membranes of most tissues and are internalized through receptor-mediated endocytosis. Once inside the cell, lysosomal enzymes hydrolyze LDL, releasing free cholesterol. In hepatocytes, LDL internalization promotes the inhibition of HMG-CoA reductase, resulting in a decrease in hepatic cholesterol synthesis (Figure 1). This process helps maintain overall cholesterol homeostasis in the body [17,18].

HDL metabolism begins with the production of nascent HDL in the liver and intestine (Figure 1). These particles mature as they incorporate CE derived from cholesterol released by peripheral tissues, allowing HDL to play a central role in reverse cholesterol transport (RCT), shuttling cholesterol back to the liver [8]. The process starts with the synthesis of its main structural apolipoprotein, apoA-I, which acquires free cholesterol and PLs from chylomicrons and VLDL as a result of LPL activity, or via the ATP-binding cassette protein A1 (ABCA1) transporter, from peripheral tissues. ApoA-I acts as a cofactor for the enzyme lecithin–cholesterol acyltransferase (LCAT), which esterifies free cholesterol on the HDL surface, converting it into CE that is incorporated into the HDL core. As HDL matures, it engages with ATP-binding cassette protein G1 (ABCG1) transporters from peripheral cells to acquire free cholesterol and interacts with cholesteryl ester transfer protein (CETP), which facilitates the transfer of TG to HDL from apoB-containing lipoproteins [19]. Through this process, HDL can either deliver cholesterol directly to the liver via the scavenger receptor class B type I (SR-BI) or indirectly transfer CE to apoB-containing lipoproteins via CETP, which are then cleared by the liver [20]. HDL can perform a specific, rapid, and unidirectional cholesterol efflux via ABCA1 and ABCG1 receptors, whereas SR-B1 carries out a slower, passive, and bidirectional cholesterol efflux [21].

## 3. Understanding HDL: Insights into Functional and Dysfunctional Particles

The Framingham Heart study was the first large epidemiological trial to evidence an inverse association between HDL-C levels and ischemic heart disease [22]. As such, for every 1 mg/dL decrease in HDL-C, the risk of coronary heart disease increased by 2% in men and 3% in women. Several experimental and in vitro approaches have attributed HDL protective effects to its capacity to promote RCT (preventing atheroma plaque formation), exert antioxidant effects, enhance endothelial function, exert cardioprotection and prevent thrombus formation [23,24,25]. Recent epidemiological studies have also revealed a U-shaped association between HDL-C levels and all-cause mortality, with very high HDL-C levels potentially increasing coronary heart diseases risk [26,27,28]. Yet, more research is required to elucidate the mechanisms behind this phenomenon and to understand why extremely high HDL-C levels may be detrimental. Likewise, it has become increasingly apparent that the presence of cardiovascular risk factors or comorbid conditions remodel HDL particles into a dysfunctional state, causing them to lose their beneficial properties and potentially become harmful [29]. In the following sections, we explore the advantages of functional HDL particles and examine the potential risks associated with altered HDLs.

### 3.1. HDL Particles: Structure and Composition

**Figure 2:**
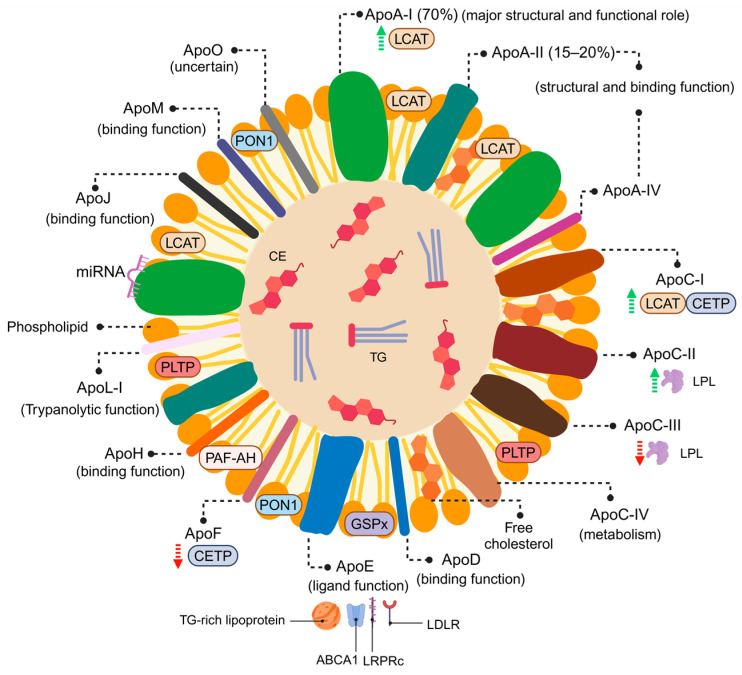
HDL structure and composition: ApoA-I constitutes 70% of the total protein content in HDL, activates LCAT, facilitates interactions with cell receptors, and has a major structural and functional role. ApoA-II represents 15–20% of the total protein and has structural and binding functions like apoA-IV. ApoC-I acts as modulator of CETP activity and as an LCAT activator. ApoC-II serves as an activator of LPL, while apoC-III inhibits its activity. ApoC-IV performs as a TG metabolism regulator. ApoD has been associated with the binding of small hydrophobic molecules. ApoE works as a ligand between TG-rich lipoproteins, ABCA1, LRPRc, and LDLR. Other apolipoproteins include apoF as a CETP inhibitor; apoH as a binder of negatively charged molecules; apoL-I as a trypanolytic factor of human serum; apoJ and apoM have been linked with the binding of small hydrophobic molecules. The function of apoO has yet to be fully elucidated [20]. The HDL lipidome is predominantly composed of PL (40–60%) and CE (30–40%), with the remainder consisting of TG (5–12%) and free cholesterol (5–10%). Moreover, HDL carries enzymes involved in lipid metabolism (LCAT and PLTP) and antioxidant activity (PON1, PAF-AH, and GSPx) [42]. HDLs have also been found to transport miRNAs that influence various cholesterol-related processes [33]. HDL: high-density lipoproteins; Apo: Apolipoprotein; LCAT: lecithin–cholesterol acyltransferase; CETP: cholesteryl ester transfer protein; LPL: lipoprotein-lipase; TG: triglycerides; ABCA1: ATP-binding cassette protein A1; LRPRc: LDL receptor-related protein; LDLR: LDL receptor; PL: phospholipids; CE: cholesterol esters; LCAT: lecithin–cholesterol acyltransferase; PLTP: phospholipid transfer protein; PON1: paraxonase 1; PAF-AH: platelet-activating factor-acetyl hydrolase; GSPx: glutathione selenoperoxidase; Green arrow: activation; Red arrow: inhibition. Created in BioRender.com.

HDL structure is characterized by a neutral lipid core composed mainly of CE and some TG, surrounded by a monolayer consisting of PLs and unesterified cholesterol that anchors various apolipoproteins [30,31]. HDLs have also been found to transport miRNAs that influence various cholesterol-related processes (Figure 2) [32,33] and other cardioprotective functions, such as inflammation regulation [34,35]. Of note, data also suggest that, beyond HDL particles, VLDL particles also carry miRNAs [36]. Over 200 different lipid species have been identified, which, together with protein variations, contribute to the high heterogeneity among HDL [30,31].

HDLs can be classified based on various characteristics; based on their shape and surface charge, they are categorized as pre-β particles or α-migrating; regarding their apolipoprotein content, particles containing only apoA-I are referred to as LpA-I, while those containing both apoA-I and apoA-II are classified as LpA-I:II; considering their size, subtypes range from the largest (HDL2a) to progressively smaller ones, including HDL2b, HDL3a, HDL3b, and HDL3c [20]; and by density, they are differentiated into dense HDL3, and less-dense HDL2 particles. Differences in composition exist within these latter HDL subfractions. As such, HDL3 particles contain lower sphingomyelin and free cholesterol levels, which enhance their fluidity and facilitate the incorporation of oxidized lipids [37], have a greater concentration of sphingosine-1-phosphate (S1P), and contain a higher proportion of proteins associated with cardiovascular protective functions (apoJ, PON1, PLTP, and PAF-AH) [38,39,40]. On the other hand, HDL2 particles are enriched with apoE and apoCs, suggesting a significant role in RCT due to their higher affinity for interacting with SR-BI and ABCG1 receptors [39]. HDL2 particles also exhibit a more pronounced vasodilatory effect, inducing greater secretion of prostacyclin (vasodilator) by endothelial cells and inhibiting the secretion of thromboxane A2 (a potent vasoconstrictor) [38]. However, excessively large HDL2 particles, with a very large diameter and high TG content, have been associated with an increased risk of coronary artery disease (CAD) [41].

### 3.2. Functional HDL Particles

#### 3.2.1. Antioxidant Properties

HDL displays antioxidant properties through apoA-I, PON1, and LCAT [43,44], which are involved in the reduction or hydrolyzation of oxidized lipids [43]. These antioxidative functions require SR-BI and ABCG1 activation [44]. HDL can remove oxidized lipids from lipoproteins and cellular membranes, avoiding endothelial cell apoptosis [45]. Additionally, HDL can prevent LDL oxidation by removing lipid hydroperoxides [43] and can inhibit LDL aggregation through interactions with hydrophobic domains, thus preventing their clustering [46].

PON1 has historically been described as an enzymatic protein capable of hydrolyzing lipid peroxides, thereby reducing the risk of atherosclerosis [47]. Conversely, other studies have shown that PON1 does not exhibit the capacity for phospholipid hydrolysis, thereby limiting its antioxidant capacity to an indirect role [48].

A direct function for apoA-I in reducing oxidative stress has also been demonstrated, along with its fundamental role in the activation of PON1 and LCAT [49]. Two methionine residues in apoA-I (Met112, Met148) are oxidized to sulfoxides during the reduction of lipid peroxides to redox-inactive hydroxides [50,51]. Additionally, CETP has been reported to facilitate the transfer of lipid peroxides from apoB-containing lipoproteins toward HDL [52], while apoM has been suggested to contribute to oxidized phospholipid binding [53].

#### 3.2.2. Vasodilatory and Antithrombotic Properties

HDLs exert multiple beneficial effects on the cardiovascular system [54]. HDLs promote vasodilation by increasing nitric oxide (NO) [55] and prostacyclin [56] production. This process begins with HDL binding to SR-B1 receptors on endothelial cells, which triggers the activation of AMPK, subsequently activating eNOS via the Akt signaling pathway [57]. Additionally, ABCG1-mediated cholesterol efflux lowers the concentration of oxidized cholesterol molecules, facilitating the activation of eNOS dimers and increasing NO production [58]. Of note, through lipid removal, HDLs also exhibit antiapoptotic and anti-inflammatory effects in the endothelium and macrophages [25,59,60,61]. A third pathway for eNOS activation involves the HDL/apoM/S1P complex, which also leads to Akt signaling activation. This pathway primarily maintains vascular integrity and further promotes cell survival and endothelial cell migration [62]. Higher NO production, in turn, leads to the reduced transcriptional activation of NF-kB in endothelial cells, resulting in a decreased expression of adhesion molecules (VCAM-1, ICAM-1, and P-selectin), pro-inflammatory receptors (TLR-2), and cytokines (MCP-1 and IL-8). In addition, HDLs may exert antithrombotic effects [63] by interacting with endothelial SR-B1 and subsequent NO release (inhibits platelet activation) [64,65] or by inhibiting thrombin-induced tissue factor expression [66]. In fact, the increase in NO also influences oxidative stress, as it acts as an antioxidant by terminating radical chain reactions and inhibiting oxidases. Additionally, NO protects against NADPH oxidase-derived reactive oxygen species (ROS) production in the vascular endothelium [67].

#### 3.2.3. Glucose Metabolism

HDLs also play a crucial role in glucose uptake and maintaining glucose homeostatic blood levels. The interaction of ApoA-I with ABCA1, ABCG1, and SR-B1 has been shown to activate the translocation of GLUT4 in skeletal muscle, endothelial cells, and adipocytes through the AMPK signaling pathway independently or synergistically with insulin [68]. This interaction induces an intracellular calcium increase and subsequent activation of CaMKK [68,69]. Several studies involving HDL infusions have reported increased GLUT4 transcript levels and a higher degree of GLUT4 translocation [70,71,72] regardless of HDL cholesterol efflux capacity [70,73]. Moreover, apoA-I binding to the β-cells membranes via ABCA1 has been described to exclude forkhead box protein O1 (FoxO1) from the nucleus and enhance the expression of *Pdx1*, a transcription factor that increases insulin secretion and regulates the expression of various genes associated with β-cell identity, functionality, and survival [74,75], besides increasing the number of insulin granules at the β-cell surface [76]. In addition, data suggest that HDLs may reduce glucagon secretion from α-cells [77].

#### 3.2.4. Others

HDL intravenous infusion has also been shown to reduce infarct size and improve cardiac function post-myocardial infarction [78,79]. The mechanisms behind this involve a reduction in apoptosis execution through the activation of RISK and SAFE signaling. The RISK pathway, which encompasses the PI3K/AKT/eNOS axis, serves to protect endothelial cells from pro-inflammatory signaling induced by TNFα, whereas the SAFE pathway is believed to be activated by S1P bound to HDL [80].

In addition to their cardiovascular protective effects, HDLs have been suggested to inhibit LPS-induced cellular activation [81], to protect against certain parasitic species [82], to regulate neural tissue formation, and to contribute to β-amyloid clearance [83]. As such, low levels of apoA-I and HDL-C have been correlated with the early onset of Parkinson’s disease [84,85] and the development of Alzheimer’s disease [86,87].

### 3.3. HDL Adverse Remodelling: The Impact of Cardiovascular Risk Factors and Comorbid Conditions in HDL Functionality

The structure and composition of HDLs are essential for binding to key receptors, which enables their primary role in RCT as well as their anti-inflammatory, anti-platelet, antioxidant, anti-apoptotic, and glucose uptake functions. Consequently, any changes to HDL can disturb their homeostasis, leading to decreased functionality and potentially transforming HDL into a harmful entity. This is particularly evident in chronic and severe conditions such as CVD [88], obesity [89], chronic kidney disease [90], liver disease [91], and diabetes [92]. Consequently, the “HDL quality over quantity hypothesis” has gained popularity as a possible explanation for missing protective effects in secondary prevention [93].

Various cardiovascular risk factors, including aging, smoking, infections, pollutants, dietary imbalances, glycation, and oxidative stress, have been shown to negatively impact HDL functionality [94]. We have reported, in highly translatable animal models by means of cardiac magnetic resonance imaging, that diet-induced hypercholesterolemia disrupts HDL function, impairing its cardioprotective [79] and atheroprotective effects [95]. These functional changes were associated with alterations in their proteomic, lipidomic, and miRNA profile [96]. Interestingly, we found that adverse remodeling of HDL can be reversed by implementing a low-fat diet and reducing LDL-C levels to a normal physiological state [97].

Hyperglycemia, one of the main features of diabetes, results in non-enzymatic glycation of plasma protein and has also been shown to adversely affect HDL composition [92]. Structural alterations in apoA-I due to glycation disrupt critical regions, impairing the activation of LCAT [98], decreasing macrophage-mediated RCT, reducing the suppression of adhesion molecule expression, and promoting ROS generation [99]. Glycation also shortens the half-life of apoA-I, further compromising HDL functionality [100]. Additionally, glycated HDL induces mitochondrial dysfunction in endothelial cells, increasing ROS levels and enhancing apoptosis [101]. Furthermore, glycated HDL exhibits a reduced ability to counteract the harmful effects of oxLDL on endothelial vasorelaxation [101].

Hypertriglyceridemia increases TG-enriched HDL levels, which have been shown to enhance net cholesterol efflux from macrophages while reducing the esterification rate due to low LCAT activity. This leads to a redistribution of cholesterol within the HDL, due to the increased TG/CE ratio, which enhances core fluidity, altering HDL lipoprotein composition [102]. These changes alter the conformation of apoA-I domains, reducing their ability to act as lipid acceptors and decreasing their exposure to the aqueous phase, thereby impairing their binding function [103] and RCT [104]. On the other hand, HDL alterations have been detected before the onset of hypertriglyceridemia in patients with MetS, including a reduction of S1P levels and subsequent impairment of eNOS activation [105,106].

Moreover, hypertriglyceridemia enhances CETP activity and reduces LPL activity [107], promoting large cholesterol ester-core-depleted HDL particles, which become targets for hepatic lipase [108], or for endothelial lipases, which hydrolyze TG as well as PLs [109]. Consequently, these modifications result in a decreased affinity for apoA-I, which reduces its plasma residence time [110] and functionality [20].

Excessive ROS levels generated by cellular lipotoxicity or by myeloperoxidase during atherosclerosis can induce structural modifications in HDL through the oxidation of the tyrosine 192 residue of apoA-I, leading to the formation of oxidized amino acid species and impairing HDL’s ability to mediate ABCA1-dependent cholesterol efflux [111,112]. Additionally, myeloperoxidase has been reported to reduce the anti-apoptotic effects of HDL, increase plaque instability [113], and diminish its antioxidant capacity by lowering PON1 activity [114]. Moreover, ROS can act on the polyunsaturated fatty acid tails of PLs or CE, generating a lipid radical that reacts with oxygen to form a lipid peroxyl radical. This oxidative molecule can propagate the reaction to adjacent PLs or polyunsaturated fatty acids, ultimately leading to HDL dysfunction [111,115].

On the other hand, genetic mutations affecting enzymes, proteins, and receptors have been shown to disrupt HDL metabolism, altering their plasma levels, modifying their composition, and impairing their beneficial properties [116]. As such, the reduction in LCAT activity, either derived from genetic mutations or exposure to pathological conditions [116], has been associated with lower HDL particle maturation and decreased HDL-C plasma levels. Likewise, genetic modifications that reduce CETP activity are associated with a decreased risk of CVD [117], resulting in increased HDL-C levels and reduced LDL-C levels. CETP-deficient individuals typically exhibit higher concentrations of HDL2a-C and enhanced SR-B1/ABCG1-mediated cholesterol efflux [118]. While these HDLs retain normal anti-inflammatory and antioxidant properties, they display reduced NO production, potentially due to lower content on S1P [119].

## 4. The Role of TG and FFAA in Metabolic and Cardiovascular Risk

According to the latest European guidelines, elevated fasting TG levels were defined as ≥150 mg/dL (≥1.7 mmol/L), moderate hypertriglyceridemia as 150–880 mg/dL (1.7–10 mmol/L), and severe hypertriglyceridemia as >885 mg/dL (>10 mmol/L). Moreover, levels exceeding 440 mg/dL (5 mmol/L) may require action to prevent acute pancreatitis, with a higher risk at levels above 880 mg/dL (10 mmol/L) [120]. A study performed in 2020 showed hypertriglyceridemia prevalence (sex- and age-adjusted) of 27.0% in the Spanish global population, 34.6% in men, and 21.4% in women [121]. In the same year, the prevalence of hypertriglyceridemia was reported in the US as 25.9%; of which, 31.6% were receiving statin treatment [122]. There are extensive data regarding the causal association between TG and the increased risk of cardiovascular events, even in patients receiving effective LDL-lowering therapy [123]. It has been observed that, in a fasting state, each 88 mg/dL increase in TG levels raises the risk of coronary heart disease by 32% in men and 76% in women [124].

**Figure 3:**
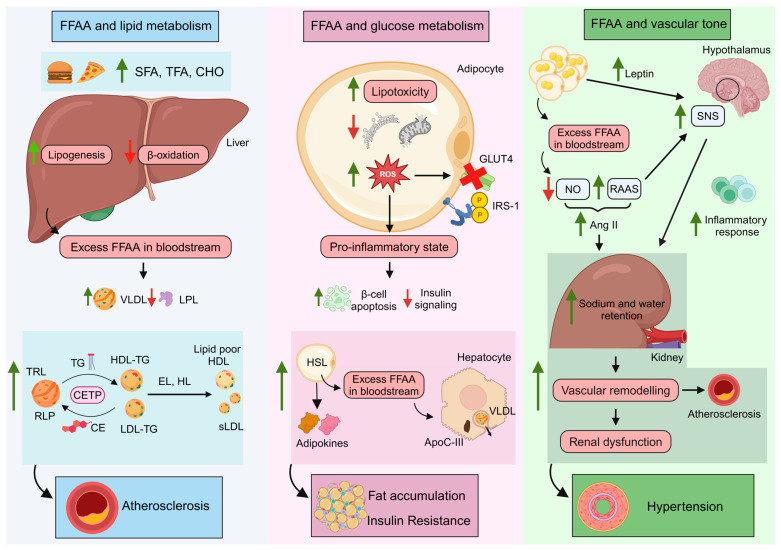
Adverse effects of high FFAA levels on lipid metabolism, glucose control, and vascular tone. (**Left**): The excessive consumption of foods rich in SFA, TFA, and carbohydrates disrupts hepatic metabolic pathways, leading to enhanced VLDL production and decreased LPL activity. This results in elevated TRL and RLP levels because of the upregulation of CETP activity. Increased CETP activity, in turn, results in lipid poor HDL and small LDL, enhancing the risk of atherosclerosis progression. (**Center**): An excess of FFAA can promote cellular lipotoxicity and generate ROS, impairing the cell’s glucose metabolism and contributing to an inflammatory state that may induce β-cell apoptosis and the development of IR. IR leads to increased secretion of FFAA and adipokines from adipocytes, stimulating VLDL and apoC-III synthesis in hepatocytes. This cascade results in further fat accumulation and exacerbates IR. (**Right**): In the case of vascular tone, an excessive release of leptin and FFAA stimulates the SNS, reduces endothelial NO production, and activates RAAS. RAAS further triggers SNS and Ang II synthesis, leading to an increase in renal sodium and water retention and promoting inflammatory cell infiltration. Collectively, these processes contribute to vascular remodeling and renal dysfunction, culminating in systemic vascular resistance and hypertension. FFAA: free fatty acids; SFA: saturated fatty acids; TFA: trans fatty acids; CHO: carbohydrates; VLDL: very low-density lipoproteins; LPL: lipoprotein lipase; TG: triglycerides; TRL: TG-rich lipoproteins; CETP: cholesteryl ester transfer protein; HDL: high-density lipoproteins; LDL: low-density lipoproteins; EL: endothelial lipase; HL: hepatic lipase; RLP: remnant lipoprotein particles; sLDL: small LDL; CE: cholesterol ester; GLUT4: glucose transporter type 4; IRS-1: Insulin receptor substrate 1; ROS: reactive oxygen species; HSL: hormone-sensitive lipase; IR: insulin resistance; ApoC-III: apolipoprotein C-III; SNS: sympathetic nervous activity; NO: nitric oxide; RAAS: renin–angiotensin–aldosterone system; Ang II: angiotensin II; Green arrow: increase; Red arrow: decrease. Created in BioRender.com.

Elevated FFAA can adversely affect lipid metabolism, glucose control, and vascular function, highlighting the importance of maintaining balanced levels of TG levels for overall cardiovascular health. As described below and in Figure 3, FFAA can stimulate the liver’s production of VLDL [125]. This process often results in elevated serum TG levels while concurrently decreasing HDL-C concentrations [126]. In addition, elevated FFAA can impair cellular metabolism and insulin action in skeletal muscle, adipose tissue, and the liver [68,127]. This impairment reduces the effectiveness of insulin in facilitating glucose uptake, which can lead to increased blood glucose concentrations. Lastly, increased FFAA can stimulate vasoconstriction and enhance sodium reabsorption in the kidneys, contributing to increased blood pressure [128].

### 4.1. FFAA and Lipid Metabolism: TG-Rich Lipoproteins and Remnant Lipoprotein Particles

A contributor to hypertriglyceridemia is a diet high in saturated fatty acids and refined sugars, among others [129]. The uptake of circulating FFAA by the liver, skeletal muscle, and other tissues is essential for lipid storage and mobilization [130]. Saturated fatty acids have been shown to reduce the expression, protein levels, and activity of hepatic LDLR, thereby decreasing LDL uptake and increasing blood LDL-C levels [131,132]. On the other hand, trans fatty acids contribute to an increase in LDL-C by reducing the catabolic rate of LDL-apoB without affecting its production levels. Trans fatty acids also increase the metabolic rate of apoA-I from HDL, without impacting its production rate [133,134]. Moreover, trans fatty acids elevate CETP activity, leading to greater TG/CE exchange between apoB-containing proteins and HDL, lowering HDL-C levels [135]. Furthermore, dietary carbohydrates serve as substrates in the liver for de novo FFAA synthesis, particularly during caloric excess. This process is amplified by glucose-induced insulin secretion, which also enhances hepatic FFAA production [136]. Excess in carbohydrate intake allows fructose to bypass the intestinal metabolism, enabling it to reach the liver directly, where it stimulates hepatic lipogenesis via SREBP1c and ChREBP. Unlike glucose, fructose metabolism does not have self-regulatory mechanisms, leading to an uncontrolled supply of substrates for lipogenesis. Additionally, fructose inhibits FFAA oxidation, contributing to higher TG hepatic levels. On top of this, fructose does not stimulate insulin production, avoiding LPL activation and reducing the clearance of TG-rich lipoproteins (TRL) [137,138].

The excess of FFAA in the bloodstream from the exogenous or/and endogenous pathway is absorbed and stored by the liver as TG [130]. This process leads to the overproduction and release of hepatic VLDL (particularly larger and TG-rich VLDL) [125], which hinders the lipolysis of chylomicrons by competing at the LPL level [139]. Additionally, hypertriglyceridemia has been shown to reduce the expression of LPL in muscle and adipose tissues [107], impairing the enzyme’s ability to bind to the lipoprotein surface, disrupting lipolysis [140] and facilitating the transport of TRL back to the liver [139]. The rise in TRL heightens CETP activity. This excessive exchange yields TG-rich LDL and HDL particles, which are hydrolyzed by lipases, producing small, dense, and pro-atherogenic LDL particles and lipid-poor HDL particles that are rapidly catabolized. This process explains the observed association between high TG levels and low HDL-C plasma concentrations [136], which contribute to the development of atherosclerosis. Specifically, increased TG levels facilitate the intimal accumulation of LDL particles, while decreased HDL-C levels—particularly the loss of HDL’s protective functions—hinder the removal of vascular cholesterol, as stated above.

On the other hand, there is an increase in the number and the half-life of TRL. The hydrolysis of TG from TRL within the endothelial lumen by LPL generates remnant lipoprotein particles (RLP). These particles are smaller than chylomicrons and VLDL, allowing them to penetrate the endothelium and reach the intimal layer. RLPs are also CE-rich molecules, as they remain a target for CETP [141]. Notably, they are considered even more pro-atherogenic than LDL-C, as they can be taken up directly by macrophages without oxidation, leading to foam cell formation and promoting the initial stages of atheroma plaque development [142]. Additionally, due to their size, RLPs can carry up to 40 times more cholesterol than LDL [143].

### 4.2. FFAA and Glucose Control

In healthy conditions, FFAA are taken up via specific membrane receptors, such as FA transport proteins 1–6 or CD36, depending on the cell type. Once inside the cell, FFAA are either stored as TG or utilized through β-oxidation as substrates for mitochondrial respiration. The binding of insulin to its membrane receptor in adipocyte cells triggers the inactivation of hormone-sensitive lipase, the enzyme responsible for hydrolyzing TG into glycerol and FFAA [144]. Moreover, insulin promotes glucose disposal in skeletal muscle cells by the translocation of GLUT4 through the activation of the IRβ/IRS-1/PI3K/Akt/AS160 pathway [145]. Under metabolic alterations, an excess of FFAA can disrupt these cellular pathways, promoting organ lipotoxicity mediated by the accumulation of TG, diacylglycerol, and ceramides [146,147]. Furthermore, it may induce endoplasmic reticulum stress and mitochondrial dysfunction, triggering the production of ROS. ROS, in turn, damages proteins involved in glucose uptake while also inducing changes in cellular homeostasis. These changes promote the activation of various intracellular pathways, including TNF-α, JNK, and PKC, among others, increasing serine over threonine phosphorylation of IRS-1, thereby impairing insulin signaling [147,148]. Moreover, oxidative stress stimulates pro-inflammatory cytokines, inflammasome activation, and macrophage tissue infiltration, resulting in cellular apoptosis. When this damage occurs in β-cells, it favors the onset of insulin resistance (IR) [127]. However, in a state of IR, hormone-sensitive lipase remains active, resulting in an increase in glycerol and FFAA and a subsequent increase in hepatic VLDL-TG secretion [149].

IR has been associated with enhanced apoC-III levels. ApoC-III hinders the binding of TRL to the cell surface, where LPL is situated. By doing so, apoC-III impairs the hydrolytic activity of LPL, as it displaces apoC-II, which is a crucial cofactor necessary for LPL to function on the lipoprotein surface. This displacement reduces the efficiency of LPL in hydrolyzing TG into FFAA, ultimately affecting lipid metabolism and leading to elevated levels of TG in the bloodstream [150]. *Apoc3* expression is partially regulated by the FoxO1 pathway, which plays a crucial role in controlling apoC-III production in the liver. Insulin facilitates the phosphorylation and subsequent inactivation of the FoxO1 pathway, resulting in the inhibition of *apoc3* gene expression and a decrease in apoC-III levels [151]. In contrast, glucose exerts an opposing effect by promoting increased apoC-III production [152]. Initially, elevated levels of apoC-III are a consequence of IR; however, as the condition advances, apoC-III itself contributes to impaired insulin signaling. This creates a feedback loop where sustained increases in circulating FFAA lead to heightened apoC-III concentrations in lipoproteins, which then remain in circulation for extended periods, exacerbating IR. On the other hand, fat accumulation in adipose tissue leads to an imbalance in TG turnover resulting in several described morpho-functional changes. These changes include alterations in adipocyte size and extracellular matrix reorganization, triggering the production of cytokines, known as adipokines (leptin, resistin, and visfatin), which have been associated with the development of IR [153,154].

### 4.3. FFAA Levels and the Vascular Tone

Recent studies have suggested that TG may act as an independent risk factor for the development of hypertension [155]. The accumulation of FFAA in renal tissues increases ROS production and impairs NO synthesis [156], which contributes to the activation of the renin–angiotensin–aldosterone system (RAAS) and, further, angiotensin II formation. Angiotensin II is a potent vasoconstrictor that stimulates the release of aldosterone and vasopressin, favoring sodium and water retention along the nephron [128,157]. Nevertheless, RAAS activates the hypothalamus, triggering sympathetic outflow towards the kidneys and promoting the activation of renal dendritic cells, which accumulate isoLG (isolevuglandins), enhancing the production of IL-6 and IL-1β [158]. This, in turn, facilitates inflammatory cell infiltration, exacerbating dysfunction and damage within renal tissue [159]. Inflammatory cells, along with cytokines and chemokines such as IL-1β, TNF-α, IFN-γ, and IL-17A, infiltrate the kidney and regulate the expression and activity of sodium transporters, leading to prolonged sodium and water retention, causing further renal damage. On top of this tissue damage, vascular remodeling occurs, characterized by the narrowing of the vascular lumen due to medial thickening and capillary loss (rarefaction) [160]. This reduction in cross-sectional area further increases systemic vascular resistance, perpetuating hypertension [161] and renal dysfunction [162].

## 5. The TG/HDL-C Ratio

Taking into consideration the key role of HDL and TG on lipid metabolism and cardiovascular risk, the TG/HDL-C ratio has emerged as a strong predictor of CAD and MetS [163,164]. The TG/HDL-C ratio has been shown to be an independent and more potent predictor of CAD than LDL-C and total-C/HDL-C or LDL-C/HDL-C ratios [164]. As such, high values of the TG/HDL-C ratio have been associated with a 2-fold increase in all-cause mortality and a 1.5- to 3-fold increase in cardiovascular events over a 5–6-year follow-up period, independent of traditional coronary risk factors and angiographic CAD severity [165,166]. Additionally, there has been an almost 2-fold increase in non-fatal myocardial infarction in intermediate-low risk patients with the highest TG/HDL-C ratio [167]. Likewise, the TG/HDL-C ratio has been considered to better identify MetS as compared to total-C/HDL-C and LDL-C/HDL-C ratios [168,169]. However, the TG/HDL-C ratio should not be used as an absolute value without considering age, ethnicity, genetics, and lifestyle [170].

## 6. Therapeutic Management to Target TG and HDL-C Levels

### 6.1. Lifestyle Changes

Exercise: The body prioritizes glucose or FFAA as the main source of energy production depending on the type, duration, and intensity of exercise. This workload is quantified in terms of VO_2max_, which is defined as the maximum oxygen consumption a person can utilize per unit of time. During low-intensity exercise, with a VO_2max_ of around 25%, energy is predominantly derived from the β-oxidation of plasma FFAA [171]. As exercise intensity increases to a moderate level, nearing 65% VO_2max_, energy demand is primarily met by FFAA released through the lipolysis of TG stored in adipose tissue. Beyond this level, classified as high-intensity exercise, there is a progressive shift toward glucose metabolism at the expense of FFAA utilization [172]. Some studies have observed that engaging in physical activity at 65–80% VO_2max_ is necessary to achieve a reduction in TG and an increase in HDL-C levels [173]. Interestingly, several studies with a large number of participants have demonstrated that prolonged intense exercise improves HDL-related cholesterol efflux [174,175]. Additionally, both acute [176] and chronic [177] exercise has been shown to enhance the antioxidant properties of HDL [174], and several studies involving patients with MetS have observed enhanced anti-inflammatory capacity associated with a decrease in TG and free cholesterol levels, alongside an increase in esterified cholesterol in HDL3b [178]. These changes correlated with greater protection against endothelial cell injury, evidenced by the reduced expression of V-CAM and decreased monocyte endothelial adhesion [179,180].

During exercise, muscle contraction leads to the release of peptides and cytokines (known as myokines) [181], including IL-6, IL-15, myostatin, irisin, and FNDCs. These myokines affect adipose tissue, particularly white adipose tissue, which serves as the primary energy storage site in the form of TG, predominantly located in intra-abdominal visceral depots [182]. Moreover, myokines activate a process known as browning, which involves the conversion of white adipose tissue into brown adipose tissue, a subtype of adipose tissue primarily responsible for energy expenditure and heat generation due to its high mitochondrial content and UCP-1 expression [182,183]. Effects on other organs have also been described, such as the pancreas, where several myokines protect β-cells from pro-inflammatory processes [184]. Furthermore, it has been reported that increased glucose uptake by the muscle is accompanied by an increase in hepatic glucose production [185], a reduction in appetite [185], the regulation of bone growth [186], and improvements in endothelial function and revascularization [187].

Diet: There is strong evidence from epidemiological studies indicating that increasing compliance with the Mediterranean diet is associated with reduced fatal and non-fatal CVD [188,189]. As such, experimental studies have proven that the intake of Mediterranean diet components preserves endothelial function [190,191], lessens platelet reactivity [192,193], reduces LDL oxidation [194], increases HDL-C levels [194], and protects against myocardial infarction damage [195,196,197]. The landmark PREDIMED trial reported a lower incidence of cardiovascular events in participants consuming a Mediterranean diet supplemented with extra virgin olive oil (EVOO) or nuts as compared to those who followed a low-fat diet [198]. Of note, participants recruited in the study presented diabetes or ≥3 risk factors (smoking, overweight or obesity, hypertension, dyslipidemia, and family history of early-onset CVD). Following these seminal findings, sub-studies were conducted to examine the effect of the Mediterranean diet on HDL functionality. An increase in cholesterol efflux was observed in those following the EVOO/nut supplementation, which was associated with an upregulation of HDL-related genes, changes in HDL-associated lipids, and improved antioxidant capacity [199]. No changes were noted in the levels of the major apolipoproteins [199]. Additionally, in the EVOO-supplemented group, an increase in LCAT activity and a decline in CETP activity were observed compared to pre-diet levels [199]. HDL also displayed enhanced antioxidant activity, which was found to be associated with an increase in PON1 content and a reduction in LDL oxidation, likely to protect LCAT from oxidative modifications [199,200]. On the other hand, HDL structural changes were also reported in the Mediterranean diet groups (particularly in the EVOO arm), showing a reduction in TG concentration and an increase in PL levels on the HDL surface. Overall, the benefits attributed to EVOO (omega-9 fatty acids) intake on HDL particles were associated with polyphenol content [201,202]. Positive effects for omega-3 fatty acids found in fish have also been described, including a reduction in TG levels [203] and an improvement in HDL beneficial effects [204].

Lycopene, a powerful antioxidant found in tomatoes, has also been associated with higher HDL-related PON1 and LCAT activity, as well as reduced serum amyloid A content and CETP activity [205]. Positive effects have also been observed with the consumption of walnuts and avocados, foods rich in polyphenols and MUFA. Walnut consumption has shown to improve HDL cholesterol removal and endothelial function [206], while avocado consumption has led to reductions in LDL-C and non-HDL-C levels, as well as a decrease in small and dense LDL particles [207]. Additionally, moderate alcohol intake (40 g of alcohol/day) has been associated with an increase in HDL-C levels, higher PON1, and reduced CETP activity [194,208,209,210,211]. Finally, beneficial effects have been observed from consuming whole grains, legumes, some vegetables, fruits, and seeds (all low-glycemic index carbohydrate) in overall CV health [212,213,214,215].

### 6.2. Drug Therapy

#### 6.2.1. Effort to Improve Primary Cardiovascular Endpoints by Increasing HDL-C Levels or HDL-Mimetics

The efficacy of CETP inhibitors has been tested in multiple phase III clinical trials in combination with statin therapy, aiming to achieve a dual positive effect on atherosclerosis by limiting its progression with statins and favoring its regression via HDL. However, most of the trials failed to meet these expectations, as we recently reviewed in Schoch et al. [23].

On the other hand, although significant improvements have been achieved in the field of HDL mimetics at an experimental level, they have consistently failed to demonstrate benefits in the clinical setting [24]. As such, a recent clinical trial administering CSL112, a human apolipoprotein A-I derived from plasma, did not show a reduction in the risk of major adverse cardiovascular events over a 90-day period [216].

#### 6.2.2. Others Therapeutic Strategies

Drug therapies for the management of TG and HDL-C levels are detailed in Table 1.

## 7. Future Perspectives and Directions

The HDL field still has several important areas that require further investigation. It is becoming increasingly accepted that HDL structure and function are better indicators of HDL’s protective function than measuring the amount of cholesterol transported by the HDL particles. Yet, it remains to be determined which specific HDL features are most strongly associated with protection against CVD to tackle them from a therapeutic perspective. In addition, while some Mendelian randomization studies have shown a causal relationship between certain HDL traits and decreased CVD risk [41,231,232], more research is needed to fully understand the genetic components influencing HDL functionality and their impact on cardiovascular health, as well as the U-shaped HDL-related behavior.

Within the TG field, determining whether TGs themselves or other components of TRLs are the primary culprit in increasing cardiovascular risk is crucial. In addition, the efficacy and safety of the new TG-lowering therapies (i.e. inhibitors of angiopoietin-like 3 protein and apolipoprotein C-III) in reducing cardiovascular events remain to be assessed in robust clinical trials. In fact, whether targeting TG production, lipolysis, hepatic clearance, or a combination of these mechanisms yields the best outcomes deserves attention. Finally, a deeper understanding of the complex interactions between HDL particles and TG within the context of MetS is essential. Investigating the molecular mechanisms that govern their biogenesis and reciprocal relationship will provide crucial insights into how alterations in these lipid particles contribute to the metabolic disease beyond providing new therapeutic targets to effectively manage MetS patients.

## Figures and Tables

**Table 1:** Drug therapies for the management of TG and HDL-C level. CETP: cholesteryl ester transfer protein; HDL: high-density lipoproteins; TG: triglycerides; MetS: metabolic syndrome; IPE: icosapent ethyl; EPA: eicosapentaenoic acid; DHA: docosahexaenoic acid; ↑: increase; ↓: decrease; CV: cardiovascular; CVD: cardiovascular disease; T2DM: diabetes mellitus type II; MACE: major adverse cardiovascular events.

Drug Class	Example(s)	Doses (Oral)	Clinical Indications (Type of Patients)	Positive Outcomes	Clinical Outcomes	Reference
**Vitamin B3**	Niacin	500–2000 mg/d	CVD, MetS	↑HDL by ~15–30%, ↓TG by ~20–50%	No effect or ↓CV events	[217,218,219]
**CETP Inhibitors**	Dalcetrapib;	600 mg/d	Recent acute coronary syndrome;	↑HDL by ~31–40%; no significant ↓TG;	No significant ↓CV events;	[220]
	Evacetrapib;	130 mg/d	Acute coronary syndrome, cerebrovascular disease, T2DM with CVD;	↑HDL by ~130%, ↓TG by ~6%;	No significant ↓CV events;	[221]
	Torceratrip	60 mg/d	High CV risk;	↑HDL by ~72%, ↓TG by ~10%;	↑CV events, ↑death;	[222]
	Anacetrapib	100 mg/d	High CVD risk, low HDL levels	↑HDL by ~145%, ↓TG by ~9%	↓CV events (men)	[223]
**Omega-3 Fatty Acids**	IPE	4 g/d	High CV risk or T2DM	↑HDL by ~3%, ↓TG by ~20%	↓CV events	[224]
	EPA + DHA	4 g/d	High risk of CVD with low HDL, elevated TG	↑HDL by ~5%, ↓TG by ~19%	No significant ↓MACE	[225]
**Fibrates**	Fenofibrate	200 mg/d	T2DM	↑HDL by ~5%, ↓TG by ~29%	No significant ↓primary outcome	[226]
	Gemfibrozil	1200 mg/d	CVD, low HDL, elevated TG	↑HDL by ~6%, ↓TG by ~31%	↓MACE	[227]
**Statin + Fibrate**	Simvastatin + Fenofibrate	40 + 160 mg/d	Low HDL, high TG	↑HDL by ~8%, ↓TG by ~26%	No significant ↓primary outcome	[228]
**Thiazolidinediones**	Pioglitazone	15–45 mg/d,	T2DM with CVD	↑HDL by ~19%, ↓TG by ~11%	↓CV events, ↓death	[229]
**Statins**	Rosuvastatin	20 mg/d	High CV risk	No significant ↑HDL, ↓TG by ~19%	↓MACE	[230]

## Abbreviations

The following abbreviations are used in this manuscript:

ABCA1	ATP-binding cassette protein A1
ABCG1	ATP-binding cassette protein G1
Apo	apolipoprotein
CAD	coronary artery disease
CE	cholesterol esters
CETP	cholesteryl ester transfer protein
CVD	cardiovascular disease
EVOO	extra virgin olive oil
FFAA	free fatty acids
FoxO1	forkhead box protein O1
HDL	high-density lipoproteins
IDL	intermediate-density lipoproteins
IR	insulin resistance
LCAT	lecithin–cholesterol acyltransferase
LDL	low-density lipoproteins
LDLR	LDL receptor
MetS	metabolic syndrome
MUFA	mono-saturated fatty acids
NO	nitric oxide
PL	phospholipids
RAAS	renin–angiotensin–aldosterone system
RCT	reverse cholesterol transport
RISK	reperfusion injury signaling kinase
RLP	remnant lipoprotein particles
ROS	reactive oxygen species
SAFE	survivor activating factor enhancement
S1P	sphingosine-1-phosphate
SR-BI	scavenger receptor class B type I
TG	triglycerides
TRL	TG-rich lipoprotein
VCAM-1	vascular cell adhesion molecule-1
VLDL	very low-density lipoproteins
